# Influence of Reduced Graphene Oxide on Effective Absorption Bandwidth Shift of Hybrid Absorbers

**DOI:** 10.1371/journal.pone.0153544

**Published:** 2016-06-07

**Authors:** Shahid Ameer, Iftikhar Hussain Gul

**Affiliations:** School of Chemical and Materials Engineering (SCME), National University of Sciences and Technology (NUST), H-12, Islamabad, Pakistan; Oregon State University, UNITED STATES

## Abstract

The magnetic nanoparticle composite NiFe_2_O_4_ has traditionally been studied for high-frequency microwave absorption with marginal performance towards low-frequency radar bands (particularly L and S bands). Here, NiFe_2_O_4_ nanoparticles and nanohybrids using large-diameter graphene oxide (GO) sheets are prepared via solvothermal synthesis for low-frequency wide bandwidth shielding (L and S radar bands). The synthesized materials were characterized using XRD, SEM, FTIR and microwave magneto dielectric spectroscopy. The dimension of these solvothermally synthesized pristine particles and hybrids lies within 30–58 nm. Microwave magneto-dielectric spectroscopy was performed in the low-frequency region in the 1 MHz-3 GHz spectrum. The as-synthesized pristine nanoparticles and hybrids were found to be highly absorbing for microwaves throughout the L and S radar bands (< −10 dB from 1 MHz to 3 GHz). This excellent microwave absorbing property induced by graphene sheet coupling shows application of these materials with absorption bandwidth which is tailored such that these could be used for low frequency. Previously, these were used for high frequency absorptions (typically > 4 GHz) with limited selective bandwidth.

## Introduction

The role of multifunctional materials as absorbers is significant in both antenna engineering and electromagnetic interference shielding/radar absorption. Several strategies have been used to minimize the effective radar cross section and decrease the reflection and emissions as well [1,2]. Surface engineering of aerospace structures is considered as an effective tool in reducing radar signatures besides other methods like structural shaping and active and passive loadings [1,3–5]. Radar-absorbing coatings have employed different kinds of materials like ceramics, polymers and polymer composites have a pivotal role in surface engineering of aerospace structures [6,7].

Microwave absorber materials are characterized normally based on the absorption mechanism, i.e., dielectric absorbers, magnetic absorber and hybrid materials [8]. Dielectric absorber materials typically involve polymers and their composites, such as epoxy, olefin, polyester, PET and LLDPE filled with carbon fiber and MWCNTs, performed well in the high-frequency region extending from 4 to 18 GHz [1,2]. Magnetic absorbers typically involve hard and soft ferrites that are normally used in relatively high-frequency region as spinel-type ferrites show Snoek’s limit, and the magnetic loss decreases drastically at high gigahertz frequency [9].

Soft ferrites, especially NiFe_2_O_4_,have been widely studied for its high microwave absorption [10]. Most of the studies involve the pristine NiFe_2_O_4_or its composite with rare earth elements; polymers; carbon nanostructures, especially CNTs; and reduced graphene oxide (rGO) for high microwave frequencies (>4 GHz) [11,12]. Additionally, the major drawback of soft ferrite such as NiFe_2_O_4_is its limited absorption bandwidth and selective absorption frequencies [13–15]. Considering these issues, NiFe_2_O_4_nanoparticles were prepared via novel modified solvothermal route and the influence of large-diameter rGO addition is analyzed by using in situ reduction and hybrid formulation utilizing the benefits of solvothermal treatment. Their magneto-dielectric properties were tailored with an aim to get high microwave absorption in the low-frequency region (mixed L and S bands) that can cover their entire bandwidth. Here, in this research work, NiFe_2_O_4_-rGO hybrid absorbers were prepared that showed excellent microwave absorption in the low-frequency region covering the entire UHF/L/S bandwidth instead of selective frequency resonances.

## Synthesis Procedure

Graphene oxide was used as a source of graphene sheets through modified solvothermal reduction process. All the chemicals used as precursors at any stage were of AR grade. Graphene oxide was synthesized using a simplified Hummer method[16];typically 3g of flake graphite was mixed with 18g of KMnO_4_ in 400ml acidic mixture (320ml:80ml H_2_SO_4_:H_3_PO_4_). The reaction mixture was stirred rapidly and continuously until the color of solution turned brown. After ceasing the oxidation, the suspension containing highly oxidized flake graphite was washed multiple times with 5M HCl and double-distilled deionized water. Nanocrystals of NiFe_2_O_4_ were synthesized via modified solvothermal approach using ethanol and DI in a definite proportion[17]. In a typical reaction, stoichiometric amounts of nitrates of both nickel and iron were dissolved in thesalt mixture separately and mixed with each other using magnetic stirring. Then NaOH solution in DI water is added to salt solution and the admixture is allowed to stir at room temperature and later transferred into autoclave vessel maintained at 180°C for 12 hrs. After the vessel temperature returned to room temperature at the completion of reaction, the residue was washed with DI water repeatedly to neutralize the pH and the later heated at 110°C to obtain the powder of NiFe_2_O_4_. Thesame method was carried out for hybrid preparation using a different weight percentage of GO powder [18].

## Results and Discussion

### X-ray Diffraction

**Fig 1:**
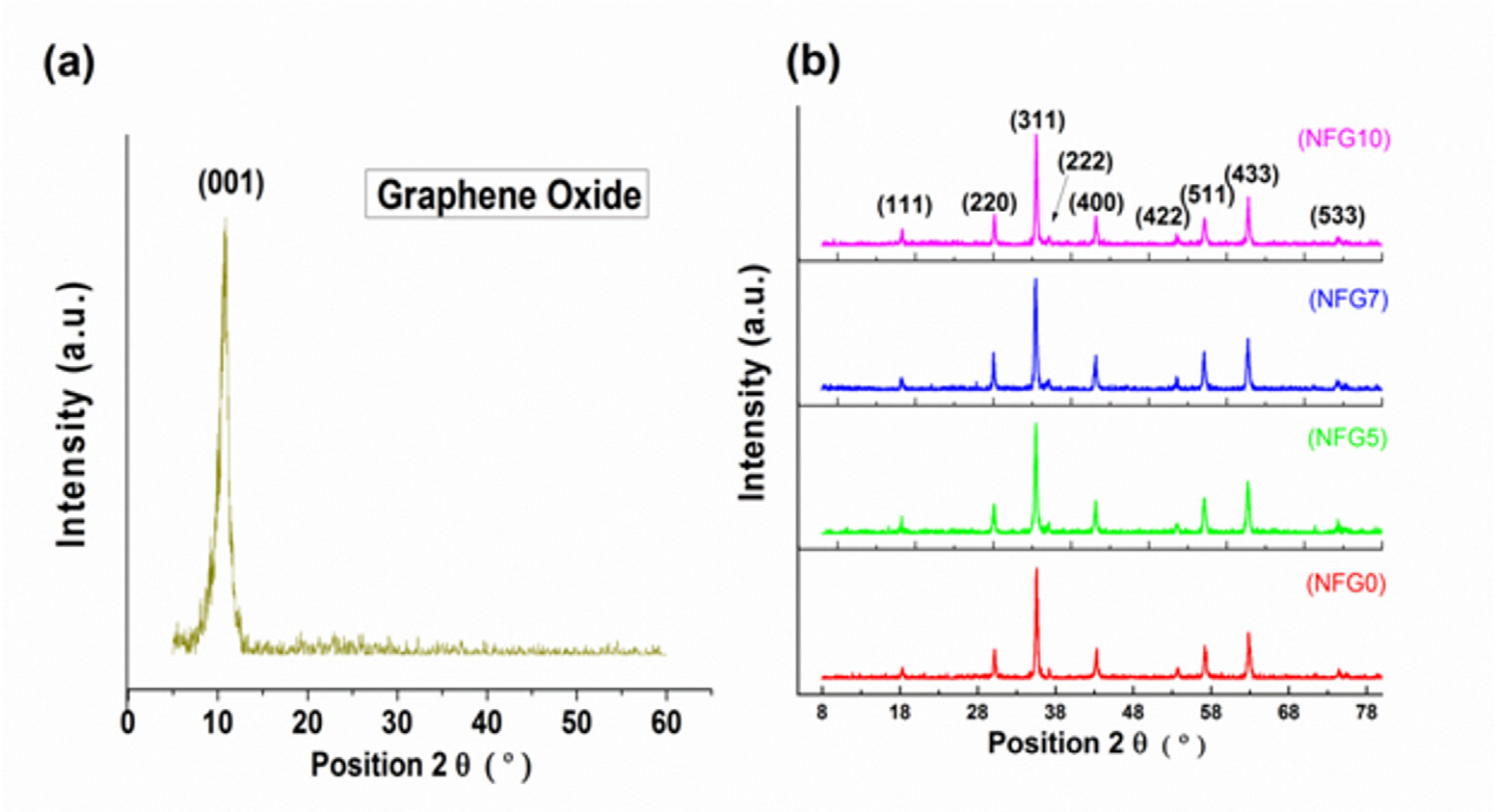
X-ray diffraction of (a) Graphene oxide (b) NiFe_2_O_4_ and composites.

X-ray diffraction (XRD) was carried out for both the NiFe_2_O_4_ nanoparticles and the NiFe_2_O_4_-rGO hybrid to analyze the structure of the synthesized nanopowder. Fig 1A shows the x-ray diffraction pattern of graphene oxide showing the characteristic peak at 10.86. The only major peak in graphene oxide pattern is due to the (001) reflection at 10.86°, which shows the complete oxidation and exfoliation of flake graphite precursor and the disappearance of reflection from major graphitic planes such as (002) and (004), which are typical of graphitic crystal structure [19,20]. The disappearance of usual graphite crystallographic diffraction peaks discussed above and emergence of peak at 10.86° shows the insertion of oxygen-bearing functional groups incurred from oxidative treatment leading to oxidized sheets of flake graphite and increased interlayer distance from the usual separation of 0.33 nm to about 0.70 nm, which is in agreement with the already reported literature[16,20]. Fig 1B shows that the as-synthesized nanoparticles of NiFe_2_O_4_ were fccstructurednanoferrites. Their structure fully matches with powder diffraction file JCPDS 10–0325. Similarly, the XRD pattern of hybrid samples NFG5, NFG7 and NFG10also agreed well with the structure of NiFe_2_O_4_. Moreover, there was no peak of graphene oxide in all the three synthesized composite samples. The absence of the characteristic peak (001) associated with graphene oxide in samples shows the complete reduction under solvothermal condition mentioned in experimental synthesis.

### Morphological Analysis

**Fig 2:**
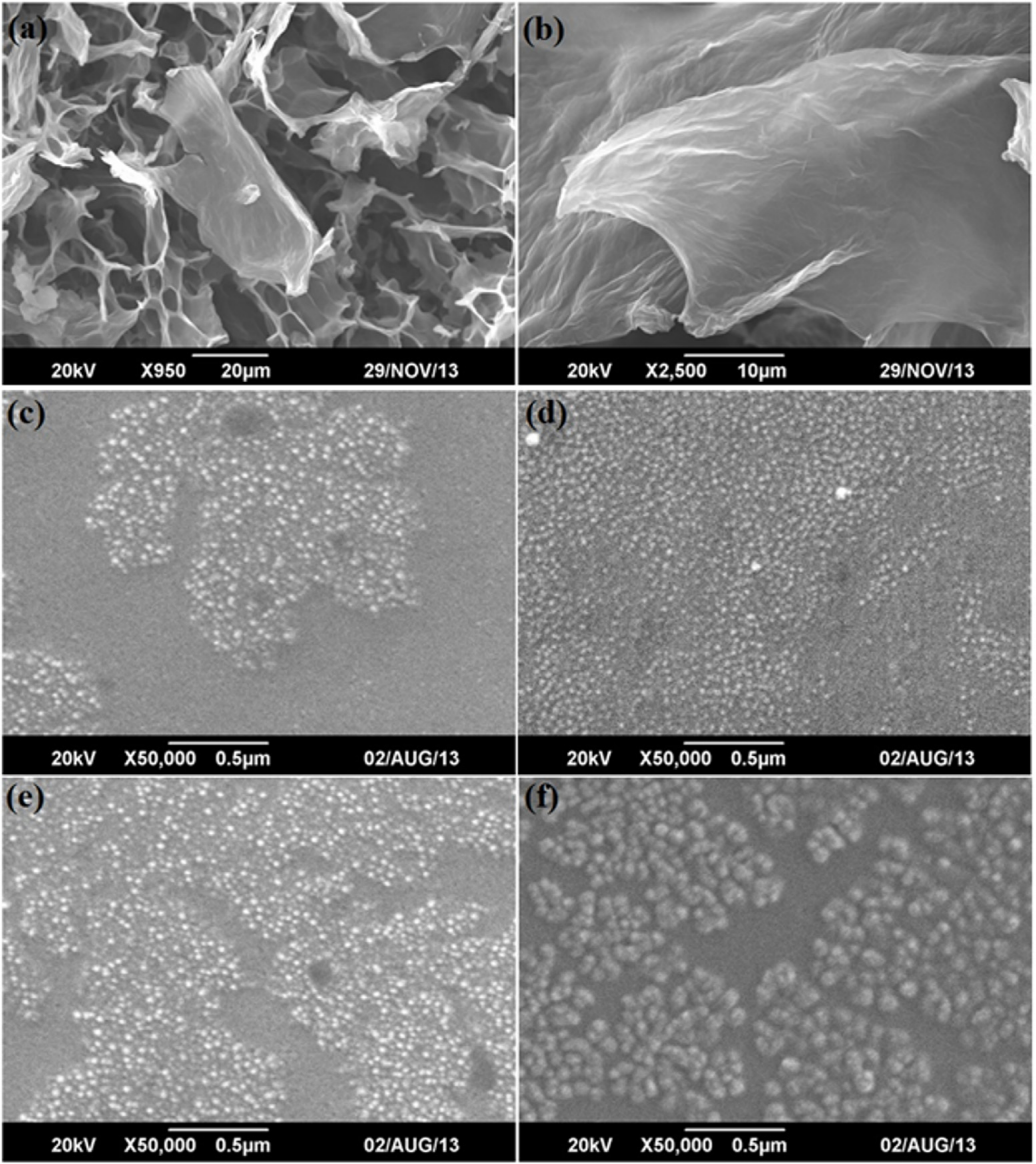
Scanning Electron Micrographs (a-b) Graphene oxide (e-f) NiFe_2_O_4_, NFG5, NFG7 and NFG10.

Scanning electron microscopy (SEM) coupled with energy dispersive x-ray spectroscopy (EDX) was used to analyze the microstructures, mainly the sizes, shapes, and distributions, and the oxidation contents of as-synthesized graphene oxide sheets, NiFe_2_O_4_and NiFe_2_O_4_-graphene composite samples NG5, NG7 and NG10, along with their chemical compositions. Fig 2A and 2B shows the SEM micrographs of freeze-dried graphene oxide sheets at two different resolutions to provide better idea of the morphology and size. It is clear from the images that the graphene sheets have sheets of large area and most of them are transparent, showing the reduced layer thicknesses, which indicates that the fair exfoliation and drying process prevented their restacking, and presenting more area for NiFe_2_O_4_to graft on. The composition of graphene oxide analyzed through EDX shows that the synthesized graphene sheets have high oxygen content (around 42%) attached with individual carbon layers. Fig 2C–2F shows the SEM images of NiFe_2_O_4_and NFG5, NFG7 and NFG10. The SEM micrographs of NiFe_2_O_4_show that the synthesized nanoparticles via solvothermal medium are spherical and have grown in a leaflike pattern with a uniform size distribution, as shown in Fig 2C. The SEM images of the NiFe_2_O_4_hybrid samples i.e. Fig 2C–2F show that the almost spherical nanoparticles have uniformly grown on the surface of graphene sheets; their composition and particle sizes are shown in Table 1.

**Table 1:** Chemical compositions with average particle sizes based upon SEM and EDX analysis.

Name	NiFe_2_O_4_(%±0.4)	rGO(%C±0.4)	Particle size(nm)
NFG0	**100**	**--**	**30**
NFG5	**93**	**07**	**31**
NFG7	**89.5**	**10.5**	**36**
NFG10	**85**	**15**	**58**

### FTIR Analysis

**Fig 3:**
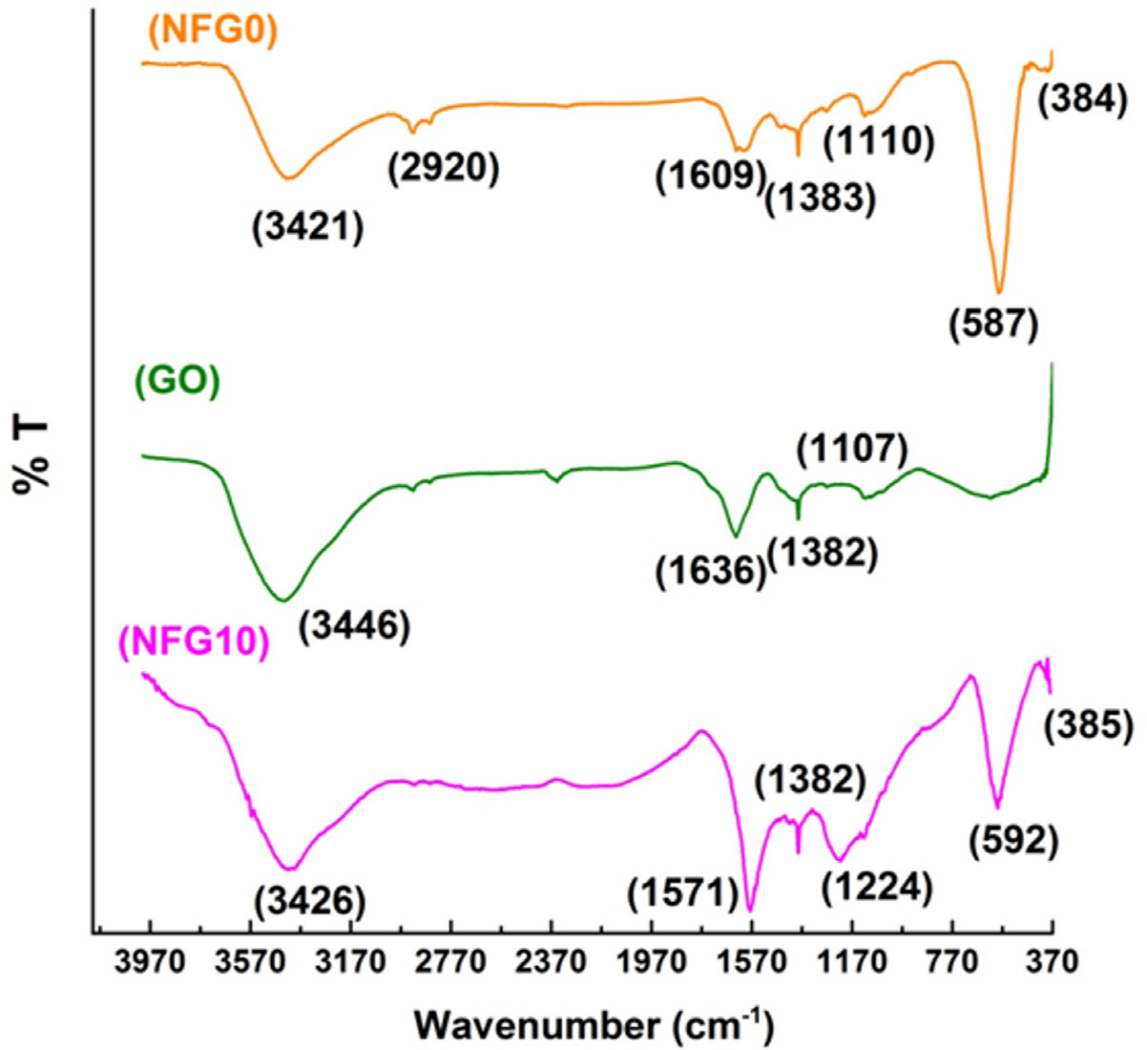
FTIR analysis of graphene oxide, NiFe_2_O_4_ and composite NFG10.

Fourier transform infrared spectroscopy (FTIR) was performed to analyze and confirm the bonding behavior, functionalization of graphene lattice with nanoferrite crystals and reduction of graphene oxide in composite. Fig 3 shows the FTIRs of the NiFe_2_O_4_ nanoparticles, graphene oxide powder and the hybrid NFG10. It is clear from the transmission spectra of NiFe_2_O_4_ nanoparticles that two majors peaks around the wave numbers 384 and 587 cm^−1^ correspond to the ν1 and ν2 bands, respectively, which are typical of ferrites[21]. The band around 384 cm^−1^ corresponds to octahedral Ni-O stretching vibrations, while the bond lying around 587cm^−1^ shows the stretching vibrations due to tetrahedral Fe-O. The peak at 1110and 1383cm^−1^ are the result of asymmetric stretching vibrations of CO_3_^2−^/NO_3_^−^ adsorbed on the ferrite matrix or of the impurities from reaction precursors[22]. The bands between and around 2800 to 3400cm^−1^ and at 1609cm^−1^ arethe result of O-H stretching and bending vibrations attached with iron ions and of moisture adsorbed on ferrite nanoparticles[12,23,24]. In case of graphene oxide transmission spectra, the major peaks are at 1636, 1382 and 1107cm^−1^. The peak at 1107cm^−1^ shows the in-plane bending vibrations of C-H bonds. The band around 1636 cm^−1^ shows the C = O stretching vibrations of the oxidized carbon backbones. The peak at 1386 cm^−1^ shows the C-OH stretching vibrations. The broad peak-like feature shown at 3446 cm^−1^ shows the presence of moisture in graphene oxide powder[16,17]. In the transmission spectra of graphene-NiFe2O4 composite material, the two bands v1 and v2 lie around 385 and 592cm^−1^. The major peak at 1571cm^−1^ shows the vibrations from oxygen-depleted carbon lattice showing the fair reduction process during solvothermal conditions. The peaks at 1224and 1382cm^−1^ appear because of CO_3_^2−^ in the composite sample[12,17,24]. Absence of the oxygen containing groups in composite sample confirms the prepared product.

## Microwave Dielectric Spectroscopy

**Fig 4:**
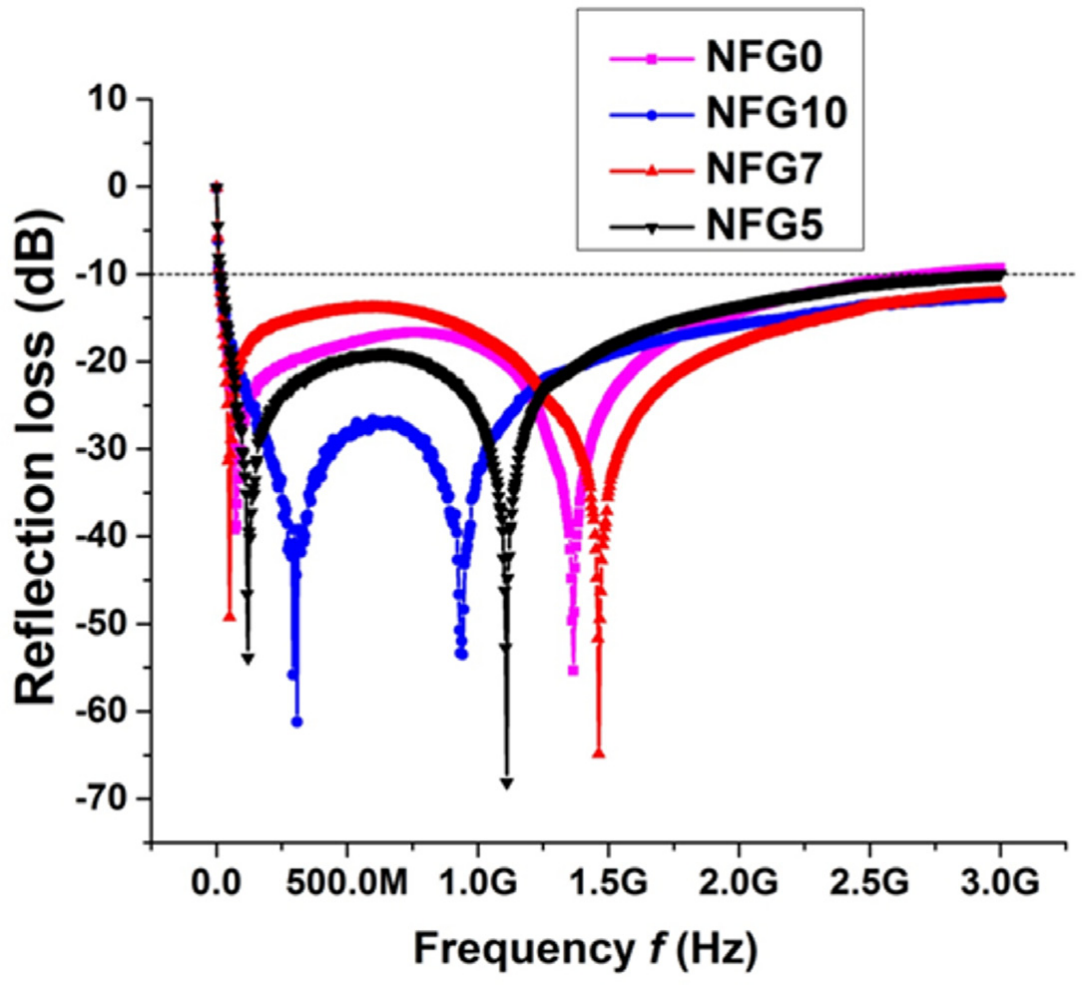
Microwave reflection loss of NiFe_2_O_4_, NFG5, NFG7 and NFG10.

Fig 4 shows microwave magneto-dielectric spectroscopy performed in mixed UHF, L and S bands ranging from 1 MHz to 3GHz. Magneto-dielectric properties, being complex variables [25,26], are expressed by the relations below:

$$\varepsilon_{r}=\varepsilon^{\prime }-j\varepsilon^{\prime \prime }$$ (1)

$$\mu_{r}=\mu^{\prime }-j\mu^{\prime \prime }$$ (2)

where ε′ and ε″ are the real and complex parts of complex permittivity (ε_r_), respectively; μ′ and μ″ are the real and imaginary parts of complex permeability μ_r_, respectively. S1 Fig and Fig 4 shows the reflection losses and the real and imaginary parts of complex permittivity of NiFe_2_O_4_ and hybrid samples NFG5, NFG7 and NFG10. Table 2 shows the dielectric data of all samples in tabular form.

**Table 2:** Dielectric properties of all samples.

Sample	1MHz					1GHz					3GHz				
	ε'	ε ''	μ'	μ''	R_L_	ε '	ε ''	μ'	μ''	R_L_	ε '	ε ''	μ'	μ''	R_L_
NFGO	4.82	0.33	0.92	-0.08	-0.19	2.19	0.11	2.84	1.36	-18.27	2.06	0.08	0.75	1.19	-9.34
NFG5	8.12	3.62	0.88	-0.03	-0.11	2.25	0.11	1.71	1.34	-26.61	2.14	0.04	0.85	1.02	-9.95
NFG7	5.57	2.37	0.94	-0.05	-0.14	1.66	0.08	2.27	1.32	-17.04	1.57	0.03	0.82	0.95	-12.18
NFG10	4.47	2.47	0.94	-0.07	-0.16	1.62	0.06	2.42	0.62	-32.14	1.56	0.02	0.92	0.54	-12.61

It is clear from the pattern of the S1 Fig that as the frequency increases, ε′ of all the samples decreases, which is identical to magnetic semiconducting nano crystals [27,28]. In case of NiFe_2_O_4_, the real parts of permittivity are 4.47, 4.82, 5.57 and 8.12 at 10MHz and these decreases to 1.61, 2.19, 1.65 and 2.24 at 1GHz for NiFe_2_O_4_, NFG5, NFG7 and NFG10, respectively. Magnetic losses are smaller than the dielectric losses in all the samples because of the dielectric polarization, which shows that the higher contribution is from dielectric loss along with the magnetic loss.

Microwave magneto-dielectric properties usually depend on a large number of factors such asthemethod of synthesizing graphene and pristine nanoferrite particles, their attachment to carbon lattice, defects in nature and distribution, sizes and shapes of graphene sheets and nanoparticles, edge structure or chirality, the nature of dielectric relaxation and the motion of electrons[2,26,27,29–31]. It is clear from S1 Fig that both the real and imaginary parts of complex permittivity decrease with an increase in frequency. Moreover, the dielectric properties increase with increasing graphene concentration and this can be explained on the basis of increased polarization and conductivity in hybrid materials NFG5, NFG7 and NFG10. Both the real and imaginary parts of complex permeability show resonance phenomenon as indicated by a hump that lies between 500 and 700 MHz and occurs in all the samples whether pristine or doped and it may be due to the spin rotation of magnetic domains in microwave frequencies, as the frequency dispersion of complex permeability is normally explained on the basis of spin and domain wall motion; at higher frequency the spin character dominates [26,32,33].The microwave absorption was evaluated by computing the input impedance and reflection losses and is indicated by the expression below:

Zin=μrεrtanh⁡(j2πfdcμrεr) (3)

RL=20log|μrεrtanh(j2πfdcμrεr)−1μrεrtanh(j2πfdcμrεr)+1|. (4)

It is clear from Fig 4 that reflection loss for all the samples NiFe_2_O_4_, NFG5, NFG7 and NFG10 are below −10dB for the entire range of frequency from 10MHz to 3GHz. There are two absorption vortexesthat appear around 200 MHz and 900 MHz for pristine NiFe_2_O_4_ nanoparticles. With the increase in graphene concentration, the vortexes for all of samples radiate away from the center absorption frequencies of pure Ni-ferrites synthesized through modified solvothermal route, with lower vortex shifting to 100, 72 and 50 MHZ and higher vortex to 1.11, 1.36 and 1.46 GHz for NFG5, NFG7 and NFG10, respectively. The results of microwave absorption shows the nanoparticles of NiFe_2_O_4_and their composites show ultrahigh performance with absorber thickness around 2 mm in the vast frequency range of 10 MHz-3 GHz as compared to the already reported absorption with high absorber thicknesses of typically 10 mm and at high frequency (>4 GHz) [10,13–15,27].

The dielectric permittivity shows the dipolar and electric polarization at the microwave spectrum and with the increases in reduced graphene oxide percentage in hybrid sample and the strained lattice causes change in electric and dipolar polarization. The change in dielectric properties can also be explained by hopping between Fe and Fe & spin/charge polarization [26]. In case of graphene or reduced graphene oxide, the basic absorption occurs through electronic spin phenomenon. As a result of reduction of graphene oxide in solution media and precipitation of ferrites on graphene sheets, the defects created also act as origin of polarization. The insertion of delocalized energy states near fermi state and electronic hysteresis causes the microwaves absorption. [32–34].

This unusually high absorption and large absorption bandwidth can be attributed to the low dimension and uniform dispersion of nanoparticles of NiFe_2_O_4_anchored with graphene sheets. This type of hybrid multilayer stacking thereby presents a continuous path for the incident energy of microwave to be absorbed via repeated magneto-dielectric losses coupled with the nanohybrid system. The reason of absorption peaks in reflection losses are mainly due to resonance phenomenon and charge hopping in ferrites based samples i.e. domain wall resonance and defects polarization centers and its coupling. [25,35]. These highly absorbing graphene-ferrite hybrid materials show promising results and can be used as commercially acceptable low-density microwave absorber. misc.

## Conclusion

NiFe_2_O_4_and its composites with large-diameter rGO sheets were prepared via solvothermal synthesis in mixed ethanol/DI media with an average particle size in the range of 30–58 nm. Microwave materials analysis shows that the both the NiFe_2_O_4_and the composite with rGO sheets show high absorbance (−10 dB) of microwave from 1 MHz to 3 GHz with absorption bandwidth comprising whole L and S bands instead of usual selective absorptions of microwaves. The maximum reflection loss was −68 dB at 1.11 GHz.

## Supporting Information

S1 Fig Microwave magneto-dielectric properties (a) real part (b) imaginary part of complex permittivity (c) real (d) imaginary parts of complex permeability of NiFe_2_O_4_, NFG5, NFG7 and NFG10.
(TIF)
