# Supplementary figures and images for: Influence of Reduced Graphene Oxide on Effective Absorption Bandwidth Shift of Hybrid Absorbers
Source: PLoS One. 2016 Jun 7;11(6):e0153544. doi: 10.1371/journal.pone.0153544 (PMC4896438; doi:10.1371/journal.pone.0153544)

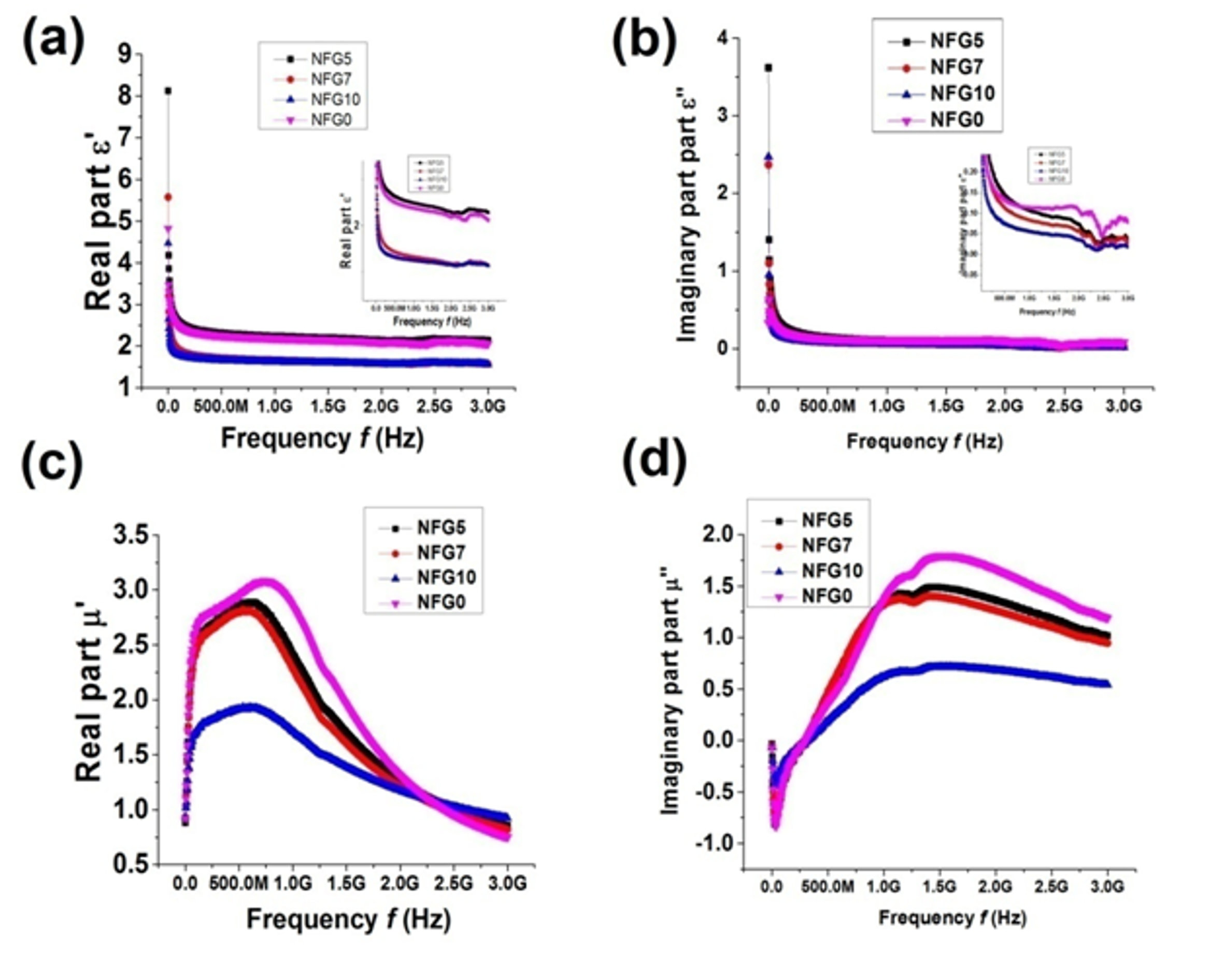

Supplement: S1 Fig — Microwave magneto-dielectric properties (a) real part (b) imaginary part of complex permittivity (c) real (d) imaginary parts of complex permeability of NiFe2O4, NFG5, NFG7 and NFG10. (TIF) [file pone.0153544.s001.tif]
